# Technical Assessment of the Targeting Accuracy of Stereotactic Frames

**DOI:** 10.7759/cureus.77091

**Published:** 2025-01-07

**Authors:** Georg A Weidlich, Neelan J Marianayagam, Robert Wiggers, John R Adler

**Affiliations:** 1 Medical Physics, Zap Surgical Systems, San Carlos, USA; 2 Neurosurgery, Stanford University School of Medicine, Stanford, USA; 3 Mechanical Engineering, Zap Surgical Systems, San Carlos, USA; 4 Radiation Oncology, Stanford University Medical Center, Stanford, USA

**Keywords:** frame rigidity, leksell frame, mechanical forces, positioning accuracy, radiosurgery headframe

## Abstract

The precision and quality of a radiosurgical procedure depend on the ability to immobilize and position the patient's head. Often, this is accomplished by means of a mechanical headframe. The ability to accurately position the head and keep it in place during the procedure depends directly on the rigidity of the headframe. This characteristic of the Leksell headframe (Elekta, Atlanta, GA, US) was evaluated by determining the impact of typical forces encountered in clinical practice on its rigidity. A quantitative analysis of the individual forces was then used to draw conclusions on the impact of the headframe's ability to accurately position the target at the desired treatment position. This article is designed to provide guidance to the user of mechanical headframes and quantitate the positioning accuracy of this frame.

## Introduction

The principles of non-invasive stereotactic radiosurgery (SRS) were developed in the 1950s by Lars Leksell [1] for the treatment of functional neurological disorders of the brain. Only subsequently did its use evolve to the focal ablation of primary benign and malignant brain tumors. Leksell’s Gamma Knife (GK) (Elekta, Atlanta, GA, US), the first-ever SRS system, utilizes accurately cross-fired cobalt-60 radiation to effect its therapeutic function. The requisite treatment precision of the GK [2] is achieved primarily via mechanical cranial immobilization, the system that requires the skeletal attachment of an external metal frame to the patient’s head. In fact, the most well-known and used frame is the Leksell stereotactic frame [3]. Once anchored to a patient’s skull, this device establishes an arc-based system of Cartesian coordinates that can, in conjunction with brain imaging, accurately locate a target of interest inside a patient’s brain while rigidly securing the head in 3D space. Although other frame-based systems have been developed for both radiosurgical and open neurosurgical procedures, the Leksell system remains the “gold standard,” especially within the field of radiosurgery.

Over the decades, “frameless systems” have also been developed to more accurately deliver focused radiation without the particular challenges of a physically attached frame [4,5]. Most linear accelerator (LINAC)-based radiosurgery platforms, i.e., robotic and gyroscopic SRS devices, have largely eschewed skeletal fixation in favor of image-guided approaches to targeting. The approach uses integrated (onboard) x-ray imaging to algorithmically correlate pre-SRS CT scans with near real-time x-rays, generating their own virtual coordinate systems within the patient treatment space. This coordinate system is then mapped to the patient's imaging data to provide accurate delivery of the stereotactic beam. To minimize patient movement between targeting x-rays, patients are routinely immobilized in custom-molded thermoplastic masks that are easily fabricated by technicians.

Although both framed and frameless SRS are employed throughout the world for the treatment of tumors and other pathologies amenable to SRS, there is scant literature on the comparative accuracy of targeting. Nevertheless, with regard to the SRS treatment of brain metastasis, both frame-based systems (e.g., GK) and frameless LINAC-based systems have been shown to achieve good results. The obvious downside of frame-based approaches is the cumbersome and modestly painful and anxiety-provoking procedure required to attach a frame to the patient’s cranium [6,7]. Moreover, pin fixation carries a small risk of hematoma or infection. In contrast, image-guided targeting is not associated with such complications; of note, during prolonged SRS procedures, headframe-based immobilization can be rather painful for patients necessitating a “break” in treatment delivery [8].

In this article, we carry out a detailed technical assessment of the Leksell type-G frame-based immobilization device, which is commonly used on the GK SRS system. The mechanical analysis of this “immobilizer” entailed a range of mechanical experiments that seemingly relate directly to all existing stereotactic frames. Herein, we present our experimental results utilizing the language of mechanical engineering. The primary goal is to provide neurosurgical practitioners with a general quantitative assessment of frame-based targeting accuracy via modern mechanical engineering analysis and thereby illuminate the strengths and weaknesses of skeletal fixation. Ultimately, these insights can refine current understandings of how best to clinically apply frame-based targeting, especially in the practice of radiosurgery.

## Technical report

**Figure 1:**
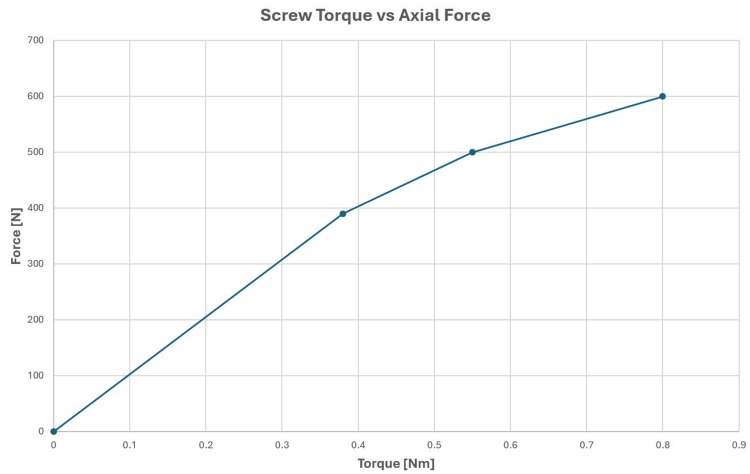
Axial force from screw torque

A range of stereotactic frames are commercially available from Radionics (Brown-Roberts-Wells (BRW) and Cosman-Roberts-Wells (CRW)) (Integra LifeSciences Corporation, Plainsboro, NJ, US), Brainlab (Munich, Germany), Fisher-Leibinger (Freiburg, Germany), and Elekta (Leksell). The function of frames is twofold: (1) define a patient-specific reference coordinate system that is used to localize a brain target and surrounding anatomy and (2) provide rigid immobilization and assist in guiding surgical (including radiosurgical) tools during a stereotactic operation. The first principles dictate that stereotactic frames must be subjected to enormous torquing forces so that their fixation by pins can provide essential stability throughout a surgical procedure. In particular, the outward-directed force applied by the tightening of attached pins is very large as shown in Figure 1.

**Figure 2:**
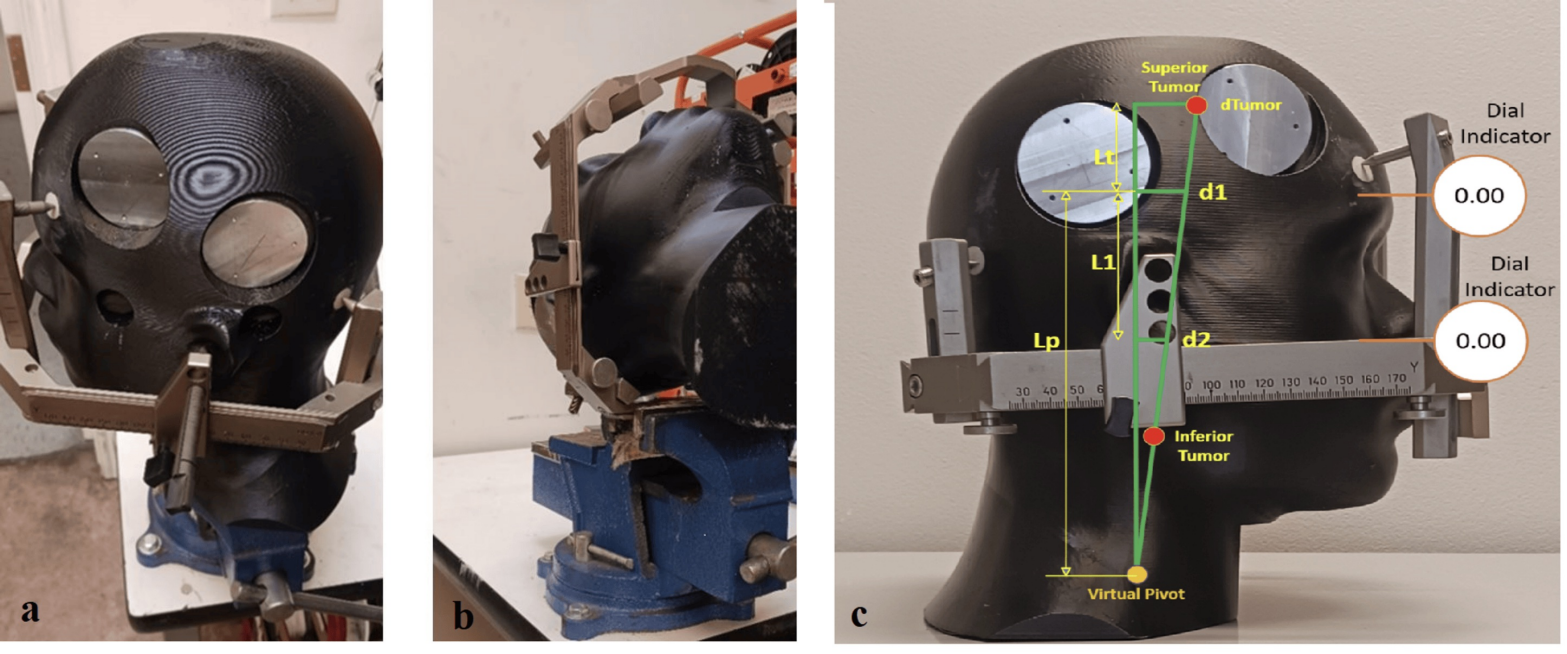
(a) Test phantom with fixation of pins; (b) phantom placed in the frame; (c) illustration of measurements and tumor locations

Given the above considerations, the Leksell type-G frame was tested for various deflections occurring in a clinical environment. A phantom was constructed of a tissue-mimicking material to model the density and composition of the human skull, while much harder inserts of high-density polymer (bone surrogate) were embedded into the phantom at the locations where frame fixation pins were placed. Figures 2a, 2b below show the phantom setup, pin fixation, and placement of the phantom inside the frame. Figure 2c illustrates the location of measurement points in relation to various tumor locations.

Ultimately, the motion of a tumor within the reference coordinate system of the headframe is of interest; additional analysis is required to convert the measured deflections into tumor motion. In this experiment, the deflections are measured linearly (dial indicators) at two locations, which are converted into both translational and angular motions. Using these two deflections along with the known distance between measurement points, a virtual pivot point can be calculated. With an angular value and a pivot point, deflections at an arbitrary distance from the pivot point can be calculated. In this experiment, the proposed superior tumor is located 5 cm more superior than the dial indicator, and the inferior tumor is located 5 cm more inferior than the dial indicator.

Figure 2c shows the geometry for trigonometry where d1 is the measured deflection (superior), d2 is the measured deflection (inferior), L1 is the distance between measurements, Lt is the distance from d1 to the proposed tumor location (given) (50 mm), Lp is the distance from d1 to the virtual pivot point (calculated), and dTumor is the calculated tumor movement.

As shown in the results, the different measurements will be used to determine the expected superior tumor and inferior tumor motions based upon this explained analytical approach. The characteristic forces that a patient’s cranium may experience during a session of framed SRS were applied. Lateral forces, torsion forces due to the rotation of the phantom in the frame, vertical drag due to a supero-inferior force, and anterior and posterior forces in the pitch direction due to patient neck extension and flexion were investigated.

The following test setup was applied:

(a) A human head phantom sized for the average (50th percentile) adult human was used with high-density inserts located where the fixation screws contact the skull. This high-density material is a reasonable surrogate for the skull. The overall rigidity of the phantom was designed to reproduce the skull characteristics as closely as possible. These characteristics included physical density, shape, and overall material consistencies such as hardness and structural integrity.

(b) A GK Leksell G-type headframe was fixated to the phantom, using the ear-locating pins.

(c) The frame was clamped in a bench vice as a surrogate for how a frame may be fastened to a radiotherapy table.

(d) Screws were tightened to a torque of 0.45 Nm (4 in-lbs) using a calibrated torque screwdriver. This corresponds to a torque typically applied in clinical practice. For the phantom setup, this resulted in an outward force of 450 N exerted by the posts onto the frame.

(e) Displacement forces were applied in 10 N increments up to 80 N maximum.

Lateral force

**Figure 3:**
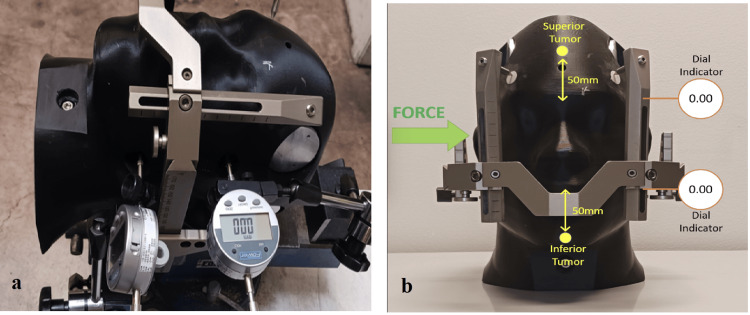
(a) Experimental set-up; (b) direction of the force

**Figure 4:**
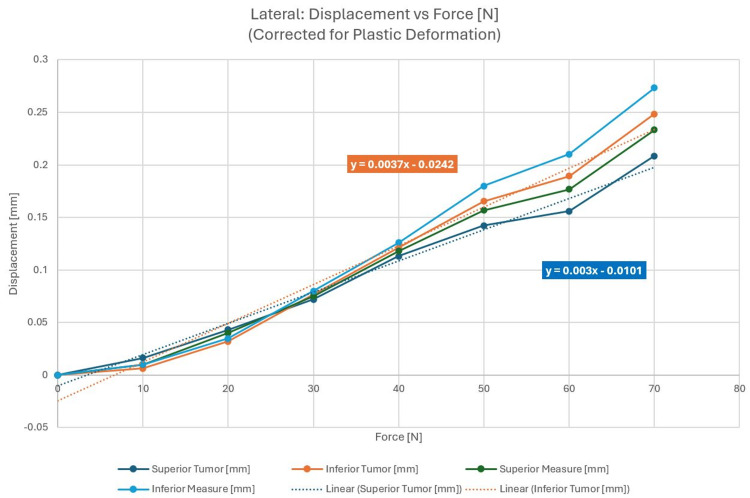
Averaged lateral displacements corrected for plastic deformation

The force was applied from the patient’s right. Two position indicators were located on the opposite sides and were distanced 80 mm apart. Figures 3a, 3b describe the experimental setup and the direction of the force. When corrected for plastic deformation, averaged displacements could be evaluated and are shown in Figure 4. Superior and inferior measurement refers to the location of the displacement measurement on the skull along the IEC y-axis (see Figure 3b). The dotted line in Figure 4 describes a linear fit of the respective displacement measurement. As a correction for plastic deformation was made, the displacement curve follows the linear fit well. The linear trendlines show phantom displacement and represent the spring constant, K, in the form y = K * x + b. Phantom surface displacements were recorded with the assumption that the target location will remain constant in relation to the phantom's surface and can be calculated based on the methodology shown in Figure 2c and described above. The objective of this study was the quantitative determination of phantom and tumor movement as a function of force exerted.

Torsion force

**Figure 5:**
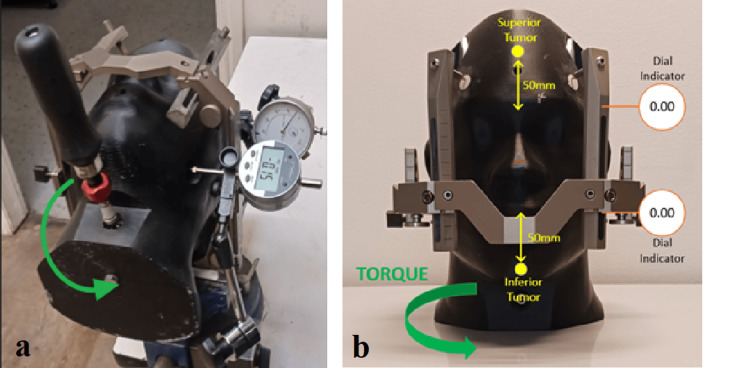
(a) Test setup for torsion; (b) direction of applied force for torsion

**Figure 6:**
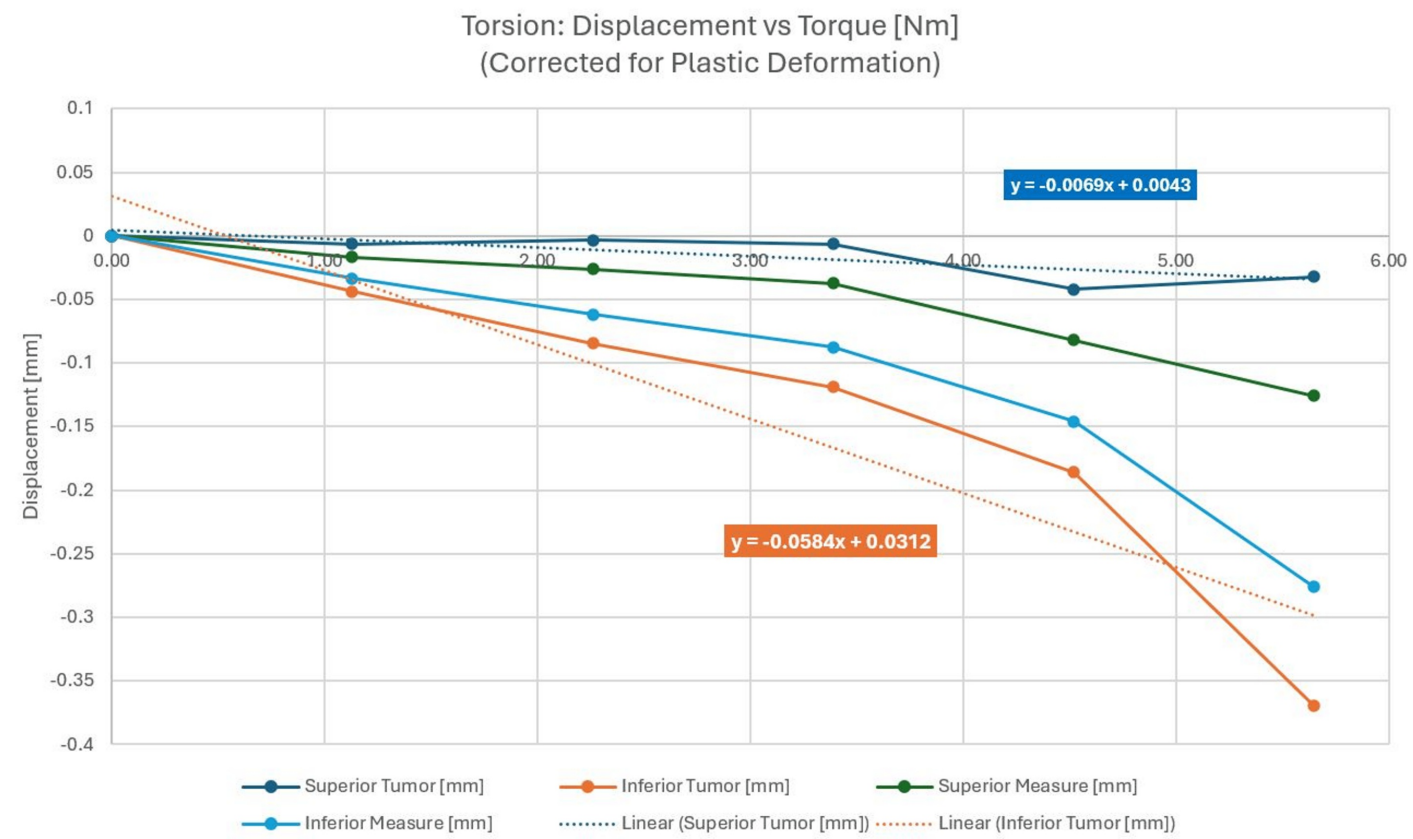
Averaged torsion displacements corrected for plastic deformation

The torque was applied at a screw located in the neck of the phantom. A calibrated torque screwdriver was used to apply a known torque. Two position indicators were located on the left side. Figure 5a shows the test setup with the displacement applied by a jawing torque and the surface gauge mounted on the phantom's left side; Figure 5b shows the application of the force. The displacement was recorded as a function of the force applied with the results shown in Figure 6. Plastic deformation corrections were made to isolate true phantom displacement.

Figure 6 clearly shows the largest displacements for the inferior measurement locations farthest from the pin fixation points. This is true for any amount of applied force and is due to the measurement point being farthest from the hinge point of the observed jaw rotation near the pins.

Supero-inferior drag

**Figure 7:**
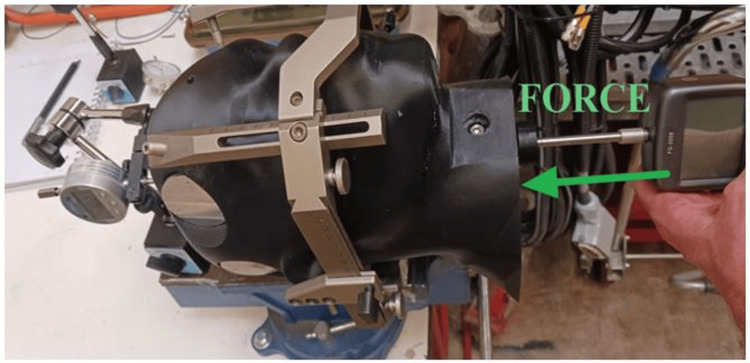
Test setup with supero-inferior force applied

**Figure 8:**
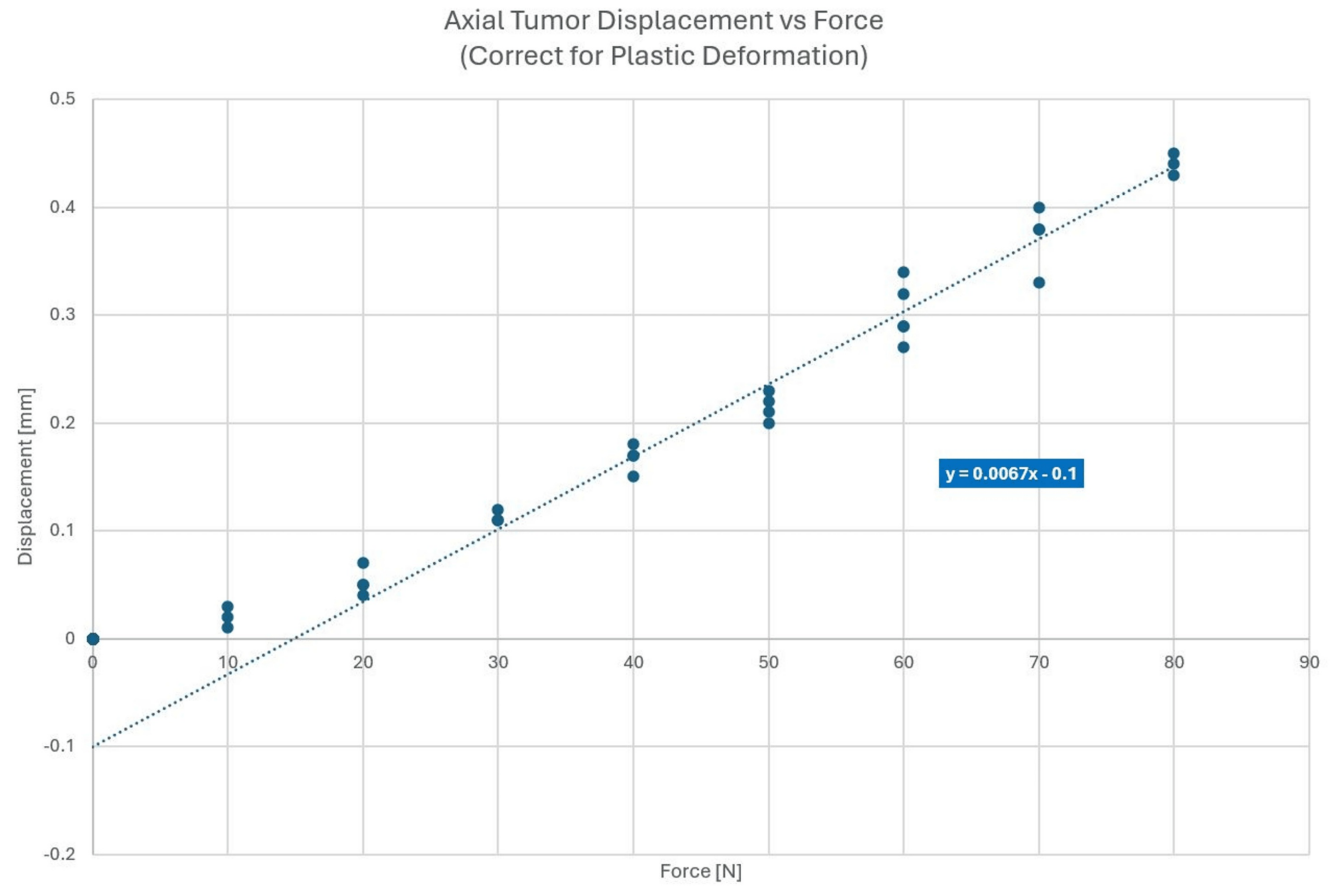
Displacement data for supero-inferior drag

Force was applied at the base of the neck in the superior direction. Figure 7 shows the experimental setup with the force applied; Figure 8 shows the associated displacements.

Supero-inferior drag is parallel to the IEC y-axis, and therefore, the results of measurements shown in Figure 8 are independent of the measurement location and were not performed at multiple locations along the y-axis.

Neck extension: anterior force

**Figure 9:**
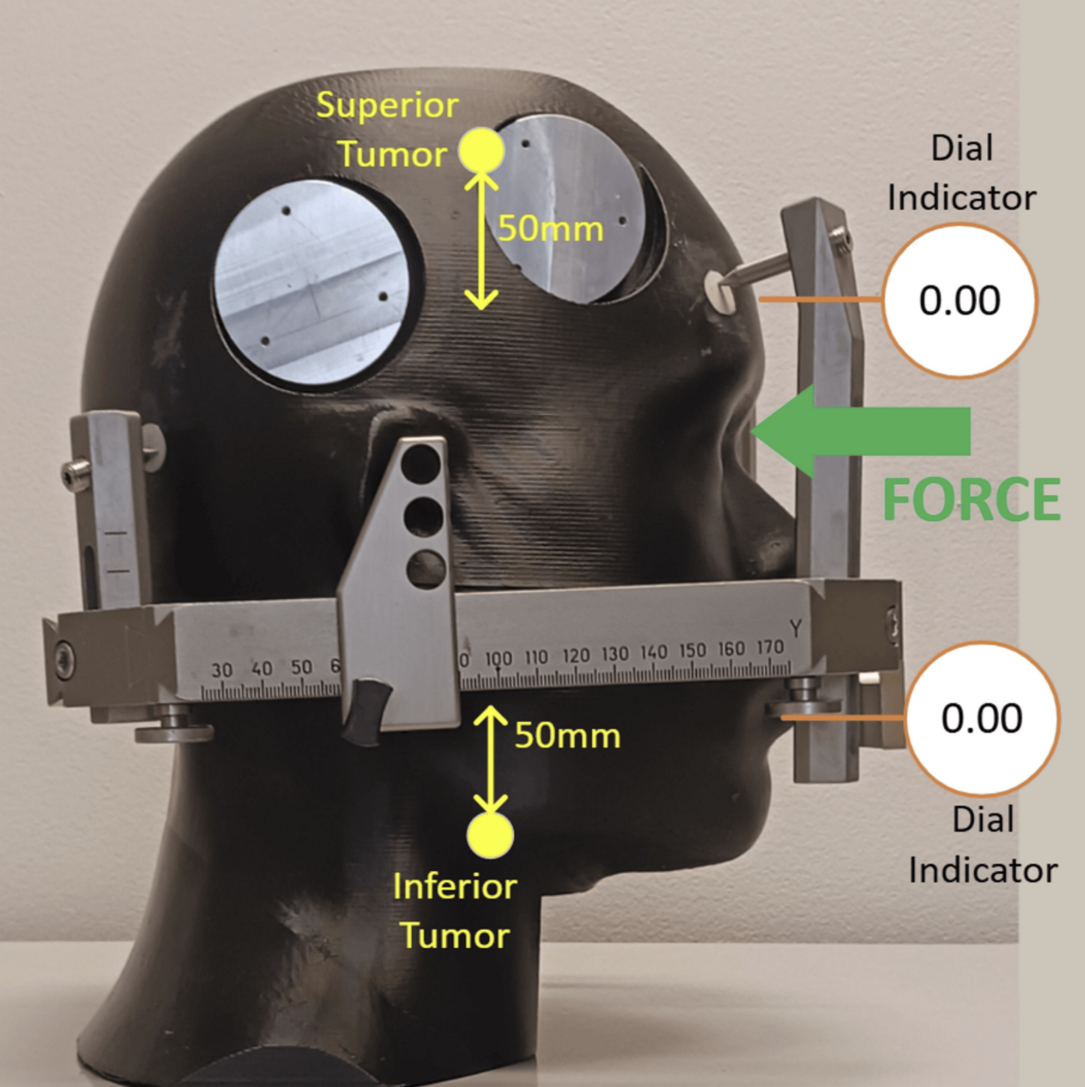
Test set-up and force applied anteriorly

**Figure 10:**
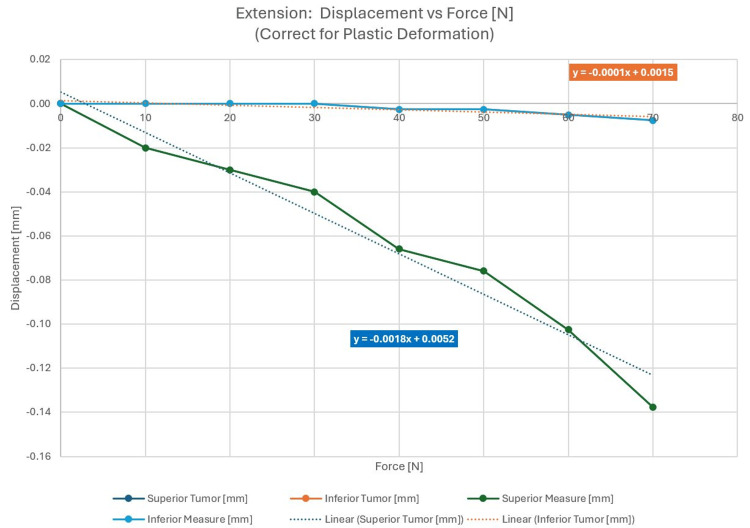
Displacement as a function of applied force, corrected for plastic deformation

External pressure was applied to the phantom anteriorly. The test setup and applied force are shown in Figure 9 with the displacement as a function of force in Figure 10.

Figure 10 shows a larger displacement in the superior region of the phantom most likely due to a posterior pitching rotation of the phantom introduced by the anterior force with a hinge point near the posterior pins.

Neck flexion: posterior force

**Figure 11:**
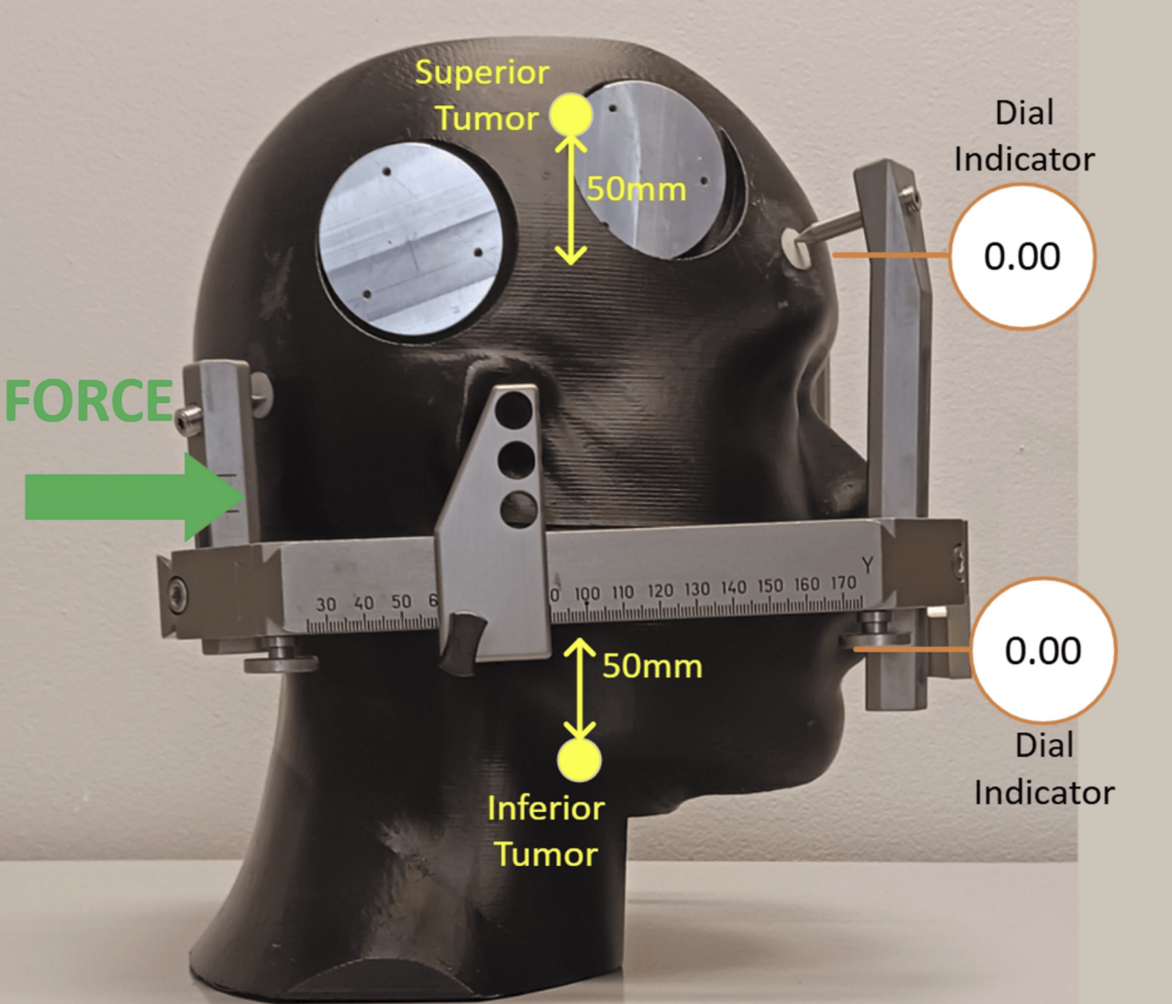
Test setup and force applied posteriorly

**Figure 12:**
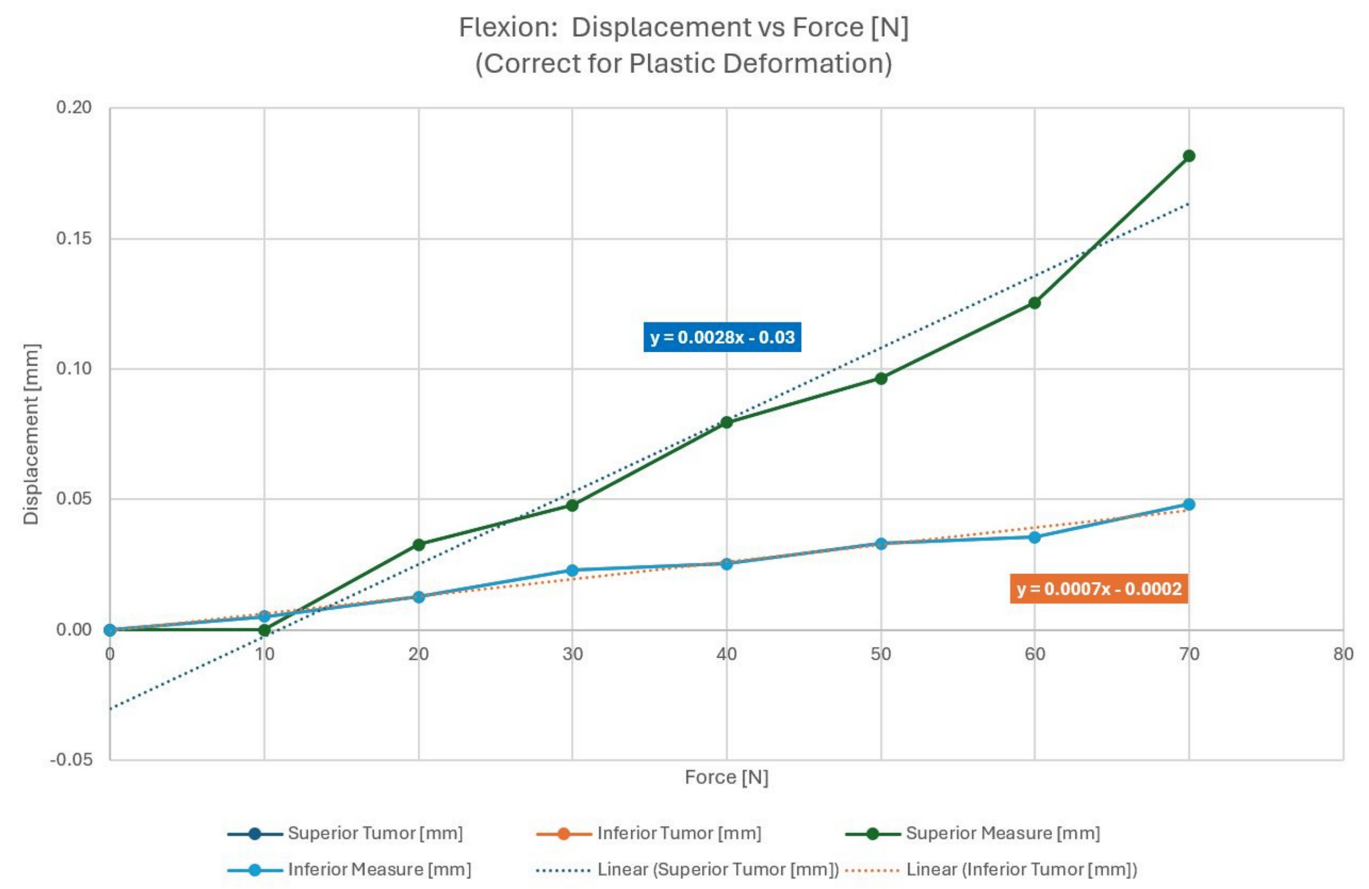
Displacement as a function of applied force, corrected for plastic deformation

In this case, the external pressure was applied to the phantom posteriorly. The test setup and applied force are shown in Figure 11. The forces are shown in Figure 12.

Application

When considering the application and force deflections, it is very important to identify the expected forces anticipated during treatment. The Leksell frame is mounted on a patient in the seated position, although imaging and treatment are done in the supine orientation where head and neck weight forces are applied to the frame. Although a patient may attempt to lie still, there may be some fidgeting or adjusting that may occur, particularly for longer treatments (>1 hour). We will not consider that the frame is mounted for up to eight hours between imaging and treatment, and during that time, the frame may be bumped or knocked while waiting for treatment.

We can infer neck strength and thus possible forces by noting that the average head (of a 75 kg person) is 5.1 kg, which converts to 50 N. Thus, the neck can certainly exert that level of force. In the extension direction, we can foresee both the nominal head weight and an exerted force. See reference [6] for anthropomorphic data and references [7,8] for neck strength data. Table 1 shows the summary of forces considered for deflection and the resulting estimated tumor movement as a function of various forces applied. Note that lateral and axial displacements are ±values as the forces may be reversed. Lateral displacement is shown as a combined result of lateral force and torsion force, axial displacement is a supero-inferior drag, and neck extension and neck flexions follow.

**Table 1:** Expected maximum tumor movement for maximum force in various dimensions

Force direction	Force	Superior tumor (mm)	Inferior tumor (mm)
Lateral	50	±0.15	±0.15
Axial (superior/inferior)	50	±0.20	±0.20
Extension	100	0.18	0.01
Flexion	40	0.08	0.03

The most notable tumor displacement is along the supero-inferior dimension. Applying the error progression law to arrive at a typical composite displacement, a value of 0.32 mm can be determined from the above data. While ±0.2 mm might be a typical displacement in the supero-inferior dimension, with a large yet feasible force, up to 0.5 mm of tumor displacement can be observed in this dimension. Any additional tumor displacement caused by plastic deformation will increase the resulting composite tumor displacement. For this phantom experiment, a plastic deformation correction as large as 0.15 mm was observed.

## Discussion

As forces are increased, material deformation follows the stress-strain curve. For elastic deformation, when the applied force results in strain less than the yield point, the material returns to its original shape. Once the strain exceeds the yield point, plastic (permanent) deflection occurs. In the frame application, the most pronounced example of plastic deformation is the screw points embedding in the plastic inserts of the phantom. This is analogous to the screws impinging on the bone of the patient’s skull.

For each directional force use case, there is a graph showing displacement corrected for plastic deformation. These graphs show the true elastic frame displacements. These are the displacements that are directly correlated to Hooke’s law of deflection (force x spring constant). This means that the frame (as a whole) also has a yield strength at which point the arms of the frame will experience plastic deformation. The required forces for this outcome were not reached in this experiment and are not expected in clinical use. The spring rate is calculated by creating a linear trendline for the elastic data points. Since the frame has a complex geometry, the spring rate changes depending on the direction of force application.

Elastic deformation occurs in line with Hooke’s law (spring-force relationship where deflections are proportional to the applied force) and is primarily associated with the metal parts within the frame (screws, extensions, primary frame, etc.). Plastic deformations are a combination of bone yield and screw-free motion. For bone yield, displacement manifests in the creation and enlargement of divots in the bone where the screw’s needlepoint contacts the skull; we see incremental displacements with each repeated force application. The screw-free motion originates from forces overcoming the internal thread friction and allows side-to-side lateral motion of the screw tip. The overall magnitudes of these displacements are directly related to the number of iterations of force applications. In clinical use, it is anticipated that there would be a low number of repeated force exertions; thus, the plastic deformation impact should be considered carefully. Instead, elastic deformations are more likely.

Upon clinical mounting of the headframe and tightening of the fixation screws, the frame has an initial plastic deformation caused by the non-linearity of the force shown as a function of applied torque in Figure 1. In the range of large forces, proportionally more torque needs to be applied to the pins to increase the “pin pressure” on the skull by a given percentage. While the phantom was designed to be as “humanoid” as possible, we do not claim that the high-density inserts in the area of pin placement mimic skull density and skull strength exactly. Therefore, any quantitative study on plastic deformation would be very specific to the material characteristics of the phantom, and it would be presumptions to draw definitive conclusions from the results applied to the plastic deformation of the patient's skull.

A maximum phantom displacement close to 0.5 mm could be observed for a large yet feasible supero-inferior applied force of 90 N (see Figure 8), especially for heavy-set patients with limited ability to lie still. Plastic deformation is expected to add to the composite displacement. This result is unexpected, especially, as the user community applies such frames with an assumed accuracy of less than 0.2-0.3 mm, depending on the vendor.

The deflections and deformations encountered in this study are alarming. Although there are multiple studies that show the efficacy of framed and frameless SRS to be equivalent, there is yet to be published a detailed report on the forces experienced by the patient during a framed session of SRS. Although these deformations are possibly clinically negligible for large targets such as tumors, this study calls into question the accuracy of frame-based SRS when treating functional disorders such as trigeminal neuralgia and essential tremor. It stands to reason that for smaller targets, even small deflections could result in greater targeting inaccuracies. Further analysis is needed in real patient settings to further quantify this phenomenon.

This study is limited to the investigation of a single-type stereotactic headframe, the Leksell G. As the exact design of other commercially available headframes differs, the results and conclusions of this study cannot blindly be applied to all such frames. However, the principles of mounting, fixation, and positioning stability are quite similar among systems available on the market. Consequently, it can be expected that overall target positioning accuracy remains comparable to the investigated Leksell headframe. A further limitation is recognized as no comparison to frameless immobilization is performed.

## Conclusions

In this paper, we have presented a detailed analysis of applied forces and their displacements during a framed SRS session. As expected, the Leksell G-Frame deflects under applied forces and during use. Given the complexity of the frame geometry, the magnitude of deflection depends upon the direction of applied forces. Overall, it is found that there are both plastic (permanent) and elastic deformations occurring in the frame/skull system due to biomechanical forces. Given the degree of deformation observed, our results cast doubt on the accuracy of framed SRS. This is especially true in cases where targets are small such as in functional disorders, and small deflections would cause greater targeting inaccuracies. More analysis is needed during live patient sessions to better determine the clinical effect.

Our results suggest that there is a greater degree of uncertainty with frame-based systems than generally appreciated and that frameless systems are more than adequate for the accurate delivery of the SRS beam without the increased risk and patient anxiety associated with framed systems.
